# Personal

**Published:** 1865-09

**Authors:** 


### PERSONAL.

Our brother and colaborer, Dr. Watt, has been very low upon a bed of sickness. So low indeed, that for a time, little or no hopes were entertained of his recovery. The disease was a low grade of fever, involving the brain and spinal cord; the lower extremities were very seriously affected. His sufferings were unutterable. The difficulties in the case were so great and camplicated, that for a considerable time, the attending physicians were wholly undecided what course to pursue. Now, we are most happy to say, he is convalescent, and ere long we hope he will be restored to complete health, though such has been the character of the disease, that a rapid recovery is not very probable, though we trust it may be otherwise.

